# Characterizing Wheat Rhizosphere Bacterial Microbiome Dynamics Under Salinity Stress: Insights from 16S rRNA Metagenomics for Enhancing Stress Tolerance

**DOI:** 10.3390/plants14071033

**Published:** 2025-03-26

**Authors:** Nourhan Fouad, Emad M. El-Zayat, Dina Amr, Dina A. El-Khishin, Haytham M. Abd-Elhalim, Amr Hafez, Khaled H. Radwan, Aladdin Hamwieh, Wuletaw Tadesse

**Affiliations:** 1 International Center of Agricultural Research in Dry Areas (ICARDA), Giza 11742, Egypt; a.hamwieh@cgiar.org; 2 Department of Biotechnology, Faculty of Science, Cairo University, Giza 12613, Egypt; elzayatem@sci.cu.edu.eg (E.M.E.-Z.); dina@sci.cu.edu.eg (D.A.); amro.osama.sa@gmail.com (A.H.); 3 Agricultural Genetic Engineering Research Institute (AGERI), Agricultural Research Center (ARC), Giza 12619, Egypt; dina_elkhishin@yahoo.com (D.A.E.-K.); haytham-mohamed@buc.edu.eg (H.M.A.-E.); 4 School of Biotechnology, Badr University in Cairo (BUC), Cairo 11829, Egypt; 5 National Biotechnology Network of Expertise (NBNE), Academy of Scientific Research (ASRT), Cairo 11516, Egypt; 6 International Center of Agricultural Research in Dry Areas (ICARDA), Rabat 10090, Morocco; w.tadesse@cgiar.org

**Keywords:** wheat, salinity, rhizosphere, microbiome, 16S rRNA, metagenomics

## Abstract

Salinity is one of the most important abiotic stress factors affecting wheat production. Salt in the soil is a major environmental stressor that can affect the bacterial community in the rhizosphere of wheat. The bacteria in the plant’s rhizosphere promote growth and stress tolerance, which vary by variety and location. Nevertheless, the soil harbors some of the most diverse microbial communities, while the rhizosphere selectively recruits according to the needs of plants in a complex harmonic regulation. The microbial composition and diversity under normal and saline conditions were assessed by comparing the rhizosphere of wheat with soil using 16S rRNA gene amplicon sequencing, highlighting the number of operational taxonomic units (OTUs). Taxonomic analyzes showed that the bacterial community was predominantly and characteristically composed of the phyla *Proteobacteria*, *Actinobacteria, Bacteroidetes*, *Firmicutes*, *Verrucomicrobia*, and *Fibrobacteres*, representing the usual microbial profile for the rhizosphere of wheat. *Idiomarinaceae*, *Rheinheimera*, *Halomonas*, and *Pseudomonas* (a strain of *Proteobacteria*), together with *Gracilibacillus* (a strain of *Firmicutes Bacilli*), were recognized as microbial signatures for the rhizosphere microbiome under saline conditions. This was observed even with unchanged soil type and genotype. These patterns occurred despite the same soil type and genotype, with salinity being the only variable. The collective action of these bacterial phyla in the rhizosphere not only improves nutrient availability but also induces systemic resistance in the plants. This synergistic effect improves plant resistance to salt stress and supports the development of salt-tolerant wheat varieties. These microbial signatures could improve our understanding of plant–microbe interactions and support the development of microbiome-based solutions for salt stress.

## 1. Introduction

Global food security is seriously threatened by population growth, climate change, and limited arable land. About 20% of the world’s irrigated cropland is affected by salt stress, which significantly reduces yields of wheat (*Triticum* spp.), an important staple food [1]. According to [2], wheat experiences hyperionic and hyperosmotic stress due to salt stress, which affects hormone balance, nutrient uptake, and overall physical growth and productivity.

Various approaches have been used worldwide to increase agricultural productivity under salt stress. These techniques include traditional breeding methods, proper selection of genotypes, introduction of desired genes, and breeding of screening genotypes [3,4]. However, these methods are costly and time-consuming. In this case, exogenous phytohormone treatment, seed priming, nutrient management, arbuscular mycorrhizal fungi, and rhizobacteria that promote plant growth are useful strategies to improve the performance of wheat under salt stress [5]. Research suggests that the rhizosphere microbiome of wheat not only adapts to adverse conditions but also has an important function in mitigating the effects of salt stress by improving plant resilience and stress tolerance mechanisms [6].

To develop effective management strategies that improve wheat performance and ensure sustainable production under challenging environmental conditions, scientists are investigating the mechanisms underlying the interaction between wheat and its rhizosphere microbiome under salt stress [7]. Previous research has shown that the introduction of plant growth-promoting bacteria into the rhizosphere of wheat can improve the plant’s ability to thrive in saline environments. This demonstrates how microbial inoculants can increase crop yields and reduce the detrimental effects of salt stress [8]. In addition, the use of biofertilizers containing beneficial bacteria such as *Bacillus* species has been shown to promote the growth and productivity of wheat even under salt stress [9]. Thus, a sustainable method to increase wheat yield in soils with high salinity has been proposed.

Furthermore, it has been shown that the integration of silicon and halotolerant bacteria into the rhizosphere can mitigate the negative effects of salt–alkali stress on wheat plants. This highlights the need to develop innovative strategies that utilize both living and non-living components to increase plant resilience to salt stress [10]. Moreover, it was also suggested that it is critical to develop innovative methods that incorporate both inanimate and living components to improve the ability of plants to endure salt stress [10]. To improve wheat production in salt-stressed areas and ensure global food security in the face of changing climatic conditions, researchers aim to exploit the beneficial interactions between wheat and its rhizosphere microbiome and implement strategic measures such as biofertilization and soil improvement.

An essential function of the plant microbiome is to improve the salinity tolerance of plants. Experiments have shown that the microbiome controls several plant stress responses, including those that help plants cope with salt-induced difficulties [11]. One possible strategy to improve plant stress tolerance under saline conditions is the degradation of *1-aminocyclopropane-1-carboxylate* (ACC) by ACC deaminase-producing bacteria in the rhizosphere [12]. In addition, studies have emphasized the importance of the rhizosphere microbiome in facilitating plant responses to environmental challenges and improving resistance to abiotic stressors [13]. By assisting in the detoxification of toxic substances, synthesis of antioxidants, and sequestration of reactive oxygen species, all of which are critical in reducing the effects of salt stress on plants, the microbiome improves plant health and resilience [14]. In addition, studies have shown that the symbiotic associations between plants and beneficial microorganisms, such as the arbuscular mycorrhizal fungus (AMF), significantly improve plant stress responses to high-salinity environments [15].

By regulating the hormonal and osmotic balance, these interactions promote plant growth and provide resistance to salt stress [16]. In addition, the root microbiota is recognized as a crucial factor for plant productivity and stress tolerance, which emphasizes the essential role of the microbiome in improving plant resistance to salt stress [17]. The plant microbiome, especially in the rhizosphere, plays a crucial role in improving plant stress tolerance by facilitating nutrient uptake, managing plant immune responses, and strengthening overall plant health and resilience [18].

Our knowledge of plant–microbe interactions has been transformed by metagenomics, which has revealed the complex interplay between plants and their microbial communities [19]. This method has shown how important these microscopic, invisible army allies play in plant health and productivity. Microbial communities have a significant impact on various aspects of plant physiology and resilience, including enhancing nutrient uptake, stress tolerance, and disease suppression [20]. In addition, metagenomics allows us to investigate the functionality of these communities. The identification of specific genes important for nutrient cycling, disease resistance, and stimulation of plant growth has been achieved by analyzing the genetic composition of plant microbiomes [21,22]. This can help to uncover the processes behind the beneficial interactions between plants and microbes [23].

The impact of metagenomics goes beyond understanding these interactions. This robust technology enables researchers to strategically regulate and structure the plant microbiome to achieve agricultural benefits. Using metagenomics, researchers can construct synthetic communities (SynComs) specifically designed to improve plant resistance, nutrient uptake, and overall productivity [24,25]. Advances in metagenomics enable precise interventions to regulate the plant microbiome, improving plant health and promoting sustainable methods of food production [26].

Recent advances in State-of-the-Art high-throughput sequencing technologies, particularly 16S rRNA amplicon metagenomics, have further revolutionized our view of microbial communities in the study of challenging complex environments such as the wheat rhizosphere microbiota. These tools allow researchers to accurately identify and quantify bacterial taxa as a function of the environment, providing insights into the response of these communities to environmental challenges such as salt stress [27].

The aim of this study was to investigate the effect of salinity on the wheat rhizosphere microbiome using 16S rRNA metagenomic analysis. Moreover, it seeks to discover microbial biomarkers associated with salt tolerance in wheat and thus adopted approaches to enhance plant resilience and productivity under salinity stress.

## 2. Material and Methods

Plant and soil Material: The genotype used in this study is Sakha-93, an Egyptian bread wheat variety (*Triticum aestivum* L.) known for its tolerance to saline conditions, from the Crops Research Institute (FCRI) field in Egypt. The soil was taken directly from the fields of the station in Giza (30.0246° N, 31.2027° E) and then refined with a 1 cm sieve. Pots with a diameter of 30 cm were used for the experiment. The study took place in the greenhouse of the Agricultural Genetic Engineering Research Institute (AGERI), Agricultural Research Center (ARC) Giza Station, Egypt.

Salinity Conditions: The temperature was regulated to simulate wheat growing season, and a photo period of 16 h of light and 8 h of darkness was maintained. The experimental design included two main treatments: normal conditions and saline conditions. In normal conditions, irrigation was carried out with tap water, while in saline conditions, artificial seawater with a molality of 200 mM was used. At the end of the experiment, the electrical conductivity (EC) of the soil was determined using a soil–water suspension at a dilution ratio of 1:3 (mass–volume) (10 g soil to 30 mL distilled water). The suspension was mixed thoroughly and allowed to stand for 30 min to ensure sufficient settling. The probe of the EC meter was then immersed in the solution, and the electrical conductivity was measured in deciSiemens per meter (dS/m) after the reading stabilized. Accordingly, the EC of a known standard molarity in the range of 25 mM to 2 M was recorded.

The soil type was categorized into bulk and rhizosphere, the latter denoting the soil that is closely associated with the plant roots. Each treatment combination (normal and saline conditions) was repeated three times for the bulk and rhizosphere soil types. A total of twelve test samples were obtained, which are labeled as follows: BC (bulk soil under normal conditions), RC (rhizospheric soil under normal conditions), BS (bulk soil under saline conditions), and RS (rhizospheric soil under saline conditions).

### 2.1. Microbial Community DNA Extraction

The soil samples were taken at a depth of 20 cm. Samples of the rhizosphere soil were taken at the same depth, taking care to remove loose soil from the root surface. Soil samples were taken at the same depth to isolate the rhizosphere and remove loose soil from the root surface. The roots were then cut and immersed in sterilized phosphate buffer (6.33 g/L NaH_2_PO_4_, 8.5 g/L Na_2_HPO_4_ anhydrous, pH = 6.5, 200 µL/L surfactant). After stirring, the roots were removed, and the extract was filtered through a sterile cell sieve with a mesh size of 100 µm. After centrifugation at 3000× *g* for five minutes, the pellet was resuspended in sterile phosphate buffer without surfactant, and the supernatant was discarded. The pellet was then reconstituted and placed in a fresh Eppendorf tube. It was then centrifuged at maximum speed for two minutes, and the supernatant was carefully removed. Samples were then stored at −20 °C until DNA extraction was performed [28].

High-quality DNA isolation was performed using the Power Soil Extraction Kit (Qiagen Cat. No. 47014) [29] according to the manufacturer’s protocol. The quantity and quality of extracted DNA were determined by electrophoretic separation on agarose gel and spectrophotometric quantification using the TECAN The Infinite^®^ M200 PRO NanoQuant instrument.

### 2.2. Metagenomics Amplicon Sequencing

The Ion Torrent S5 platform (ThermoFisher, Waltham, MA, USA) and the Ion 16S™ Metagenomics Kits (Thermo Fisher Scientific, Waltham, MA, USA) were used to perform 16S rRNA-targeted metagenomics deep sequencing according to the manufacturer’s instructions.

#### 2.2.1. 16S rRNA Gene Amplification

Targeted sequencing assays were performed using two primer pools for amplification by PCR reactions of the hypervariable regions of the 16S rRNA gene (V2, V4, and V8) in one reaction pool and (V3, V6–7, and V9) in a second reaction pool using the Ion 16S™ Metagenomics Kit. Reactions were checked for the presence of amplification bands using gel electrophoresis. Pool 1 and Pool 2 of each sample were then combined to proceed to the next steps.

#### 2.2.2. Library Preparation and Next-Generation Sequencing

Using Agencourt AMPure Beads (Beckman Coulter, Brea, CA, USA), the combined PCR products were then purified according to the manufacturer’s guidelines to remove impurities. Library preparation was then continued to ligate adapters and barcodes after final repair. These were added separately to the library fragments of each sample before the final step of library enrichment to further increase yield. Prior to pooling, all libraries were quantified using a TaqMan-based qPCR reaction for normalization, which was mainly used to mix an equimolar amount of each library. Subsequently, high-throughput next-generation sequencing was then performed according to the guidelines of the Ion Torrent S5 system.

### 2.3. Sequence Analysis

The generated data were demultiplexed, and the reads were filtered and trimmed for adaptors using the *Torrent Suite* (Thermo Fisher Scientific, Waltham, MA, USA). Reads were then aligned and mapped using Thermo Fisher Scientific’s *Ion Reporter™* Software v5.10 (Thermo Fisher Scientific, Waltham, MA, USA) with default parameters for the metagenome analysis pipeline based on the curated Greengenes and premium curated MicroSEQ ID databases. The filtered sequence reads *(Phred ~ Q20)* was used to determine operational taxonomic units (OTUs) using the *QIIME Suite software tools* (version 1.8, University of Colorado, Boulder, CO, USA).

#### Data Filtering and Normalization

A low-count filter with a minimum count of 10 was used to remove features with very low frequency. A prevalence filter with a threshold of 20% was also used to remove less common traits and focus the analysis on taxa that are consistently present in a large proportion of sites. In addition, we applied a low variance filter with a 10% threshold; all variables below this percentage were discarded to improve the detection of biologically significant signals. This is to ensure that the observed variations in microbial abundance between treatments were not simply the result of unequal sequencing attempts. Rarefaction was performed to normalize each sample to the minimum library size (excluding technical replicates) to minimize bias due to differences in sequencing depth. Cumulative sum scaling CSS was used to account for differences in sequencing depth between samples to ensure comparability of data.

### 2.4. Visual Exploration Profiling

**Figure 1:**
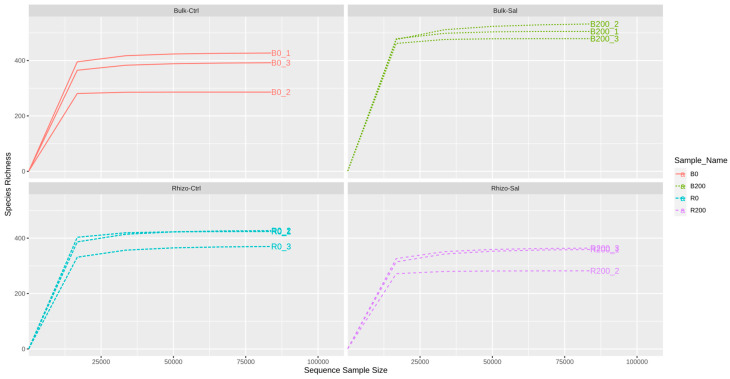
The rarefaction curve shows the relationship between the number of OTUs (operational taxonomic units) and the number of sequences per sample. As the number of sequences increases, the curve reaches a plateau, meaning that additional sequencing results in fewer new OTUs.

Rarefaction curve analysis was performed with the modified function *ggrare* from the package *ranacapa*) [30]. This was conducted to test whether the reads in a sample are sufficient to reach a plateau, meaning that as the number of sequences increases, the gain in newly discovered OTUs is limited (Figure 1).

The taxonomic composition of the community was visualized by a direct quantitative comparison of abundances at the level of phylum, family, and class taxa. Samples were grouped into four sample types: soil types and salinity types. The top 10 taxa were displayed based on the total sum of their numbers in all samples. The percentage of relative abundance was displayed as part of a stacked bar chart.

### 2.5. Community Profiling

#### Alpha, Beta Diversity, and Core Microbiome

An alpha diversity analysis was performed using the *phyloseq* package to measure diversity within the community. Four indices were used to calculate alpha diversity. The richness of the community was assessed using the *Observed* and *Chao1* indices in relation to the total number of species/taxa. In addition, evenness was calculated using the *Shannon* and *Fisher* indices to examine abundance along with the number of species/taxa.

To gain a comprehensive understanding of the diversity and composition between samples, a beta diversity analysis was performed. This involved analyzing the presence, absence, or abundance of different taxa within the dataset. Beta diversity analysis was performed using the *phyloseq* package. We used three different analytical methods to comprehensively assess the differences in microbial composition between samples. Comparison between samples was facilitated by calculating dissimilarities or distances using non-phylogenetic methods such as Bray–Curtis distance and Jensen–Shannon divergence.

Method 1 used principal component analysis (PCoA) as the ordination method and the Bray–Curtis index as the distance metric, both of which were applied at the feature level of taxonomic classification. Statistical analysis was performed using PERMANOVA, which allowed us to detect significant differences in microbial community composition between groups.

Method 2 was similar to method 1, but the Jensen–Shannon divergence was used as the distance method and ANOSIM as the statistical method.

Method 3 used non-metric multidimensional scaling (NMDS) for ordination and retained the Bray–Curtis index for feature-level distance calculations, using PERMANOVA for statistical analysis [31].

The core microbiome was also examined to identify taxa or traits that were stable in the microbial community composition across the microbial community of all four treatments: BC, BS, RC, and RS. This approach helps to understand the fundamental components that remain stable in the microbiota. When performing the core microbiome analysis, we established two crucial parameters. The first parameter, called sample prevalence, defines the minimum proportion or percentage of samples in which a taxa or trait must as the threshold be present to be considered part of the core microbiome. A threshold value of 20% was set for sample prevalence. The second parameter, relative abundance, defines the proportion of the occurrence of a taxa or trait in the samples that qualifies it as a core member. In our analysis, a relative abundance threshold of 0.01% was used. The analysis of the core microbiome was performed using the web-based tool microbiome analyst.

### 2.6. Interactions Through Spearman Rank Correlation Analysis

Spearman’s rank correlation coefficient (rho) was used as a statistical method to assess the strength and direction of linear relationships between microbial traits in different samples at strain and family levels. For this purpose, the Microbiome Analyst tool was used for this purpose (available at: http://www.microbiomeanalyst.ca (accessed on 1 June 2023)). It provides predictions about patterns that are co-occurring or mutually exclusive within our microbial community.

### 2.7. Differential Abundance Analysis

The differences between the two groups in the relative abundances of taxa from the phylum to the species level were assessed by analyzing the comparison of the microbiome *MaAsLin2* models. The significant results of this analysis were validated with generalized linear models implemented in multivariate association models with linear models (*MaAsLin2*, negative binomial model). Analyses were performed using *R* (version 4.0.2; R Foundation for Statistical Computing, Vienna, Austria). The taxa-wise false discovery rate (FDR > 0.05) adjusted *p*-value using the Benjamini–Hochberg adjustment for the multiple testing correction method and the Log2FC level ≥ 2.

## 3. Results

### 3.1. Microbial Abundance Data

A total of 12 samples and 1110 traits or taxa were available in Table 1. The dataset was processed with GreengenesID for OTU annotation. Overall, 752 OTUs had a count of two or more. The study included three experimental factors with replicates, each of which was a discrete variable. The total number of reads across all samples was 4,526,902, with an average of 377,241 per sample. The highest number of reads observed in a single sample was 796,235, and the lowest was 141,355.

### 3.2. Visual Exploration

**Figure 2:**
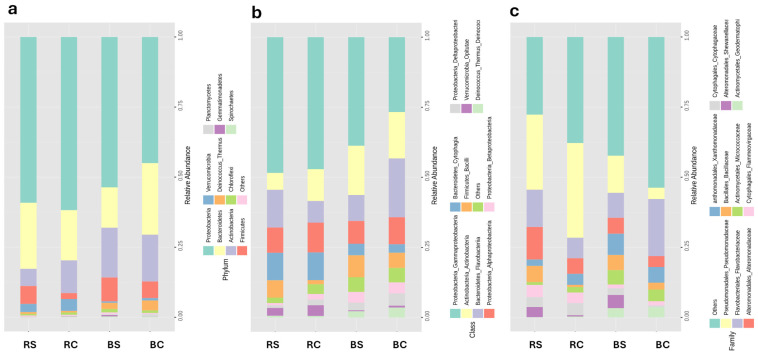
Abundance profiling at phylum, class, and family level. The stacked bar chart illustrates the relative abundances of the different taxa in the samples at three taxonomic levels: (**a**) phylum, (**b**) class, and (**c**) family. BC: Bulk under normal conditions. BS: Bulk under saline conditions. RC: Rhizosphere under normal conditions. RS: Rhizosphere under saline conditions.

Looking at the broader framework at the phylum and class level, there is a shift in the relative abundance of microorganisms between the bulk and rhizosphere under saline conditions (BS, RS) and their counterparts under normal conditions (BC, RC) (Figure 2a,b). Under saline conditions, the strain of *Proteobacteria* accounts for 54% of the bulk soil (BS) and 59% of the rhizosphere soil (RS), compared to 45% in the bulk soil (BC) and 62% in the rhizosphere soil (RC) under normal conditions. Within this strain, *Gamma-Proteobacteria* increased from 27% to 39% in the main soil (BS) under saline conditions, while they remained relatively stable in the rhizosphere soil (RS), with a slight increase from 47% to 48% after salt treatment.

However, *Alpha-Proteobacteria*, *Beta-Proteobacteria,* and *Delta-Proteobacteria* showed a slight decrease under saline conditions. For *Bacteroidetes*, although the overall percentage decreased from 24% to 13% in (BS), it was interestingly recruited by the plant and increased from 18% to 23% in (RS). Notably, *Flavobacteriia* decreased from 21% to 9% in (BS), but remarkably, it was recruited and increased from 8% to 13% in (RS). *Actinobacteria* remained relatively stable in (BS) (17% to 18%) but decreased significantly from 11% to 6% in (RS). *Deinococcus_Thermus* (*Deinococci*) and *Firmicutes* (*Bacilli*) show slight decreases in (BS), with *Bacilli* increasing from 2% to 6% in (RS). *Verrucomicrobia* (*Opitutae*) decreased under saline conditions from 1% to 0% in (BS) and from 4% to 3% in (RS).

At the family level (Figure 2c), (BC) was dominated by *Flavobacteriales_Flavobacteriaceae* (20%), while abundance decreased to 9% in (BS). In RC, *Pseudomonadales_Pseudomonadaceae* were significantly more abundant (34%), and this dominance continued under saline conditions in RS at 27%. *Alteromonadales_Alteromonadaceae* increased from 6% to 12% in (RS) under saline conditions, as did *Bacillales_Bacillaceae* from 1% to 6%. The analysis showed that the families *Pseudomonadales_Pseudomonadaceae* and *Alteromonadales_Alteromonadaceae* have adapted to the saline conditions.

### 3.3. Microbial Community Profiling

#### 3.3.1. Alpha Diversity Assessment

**Figure 3:**
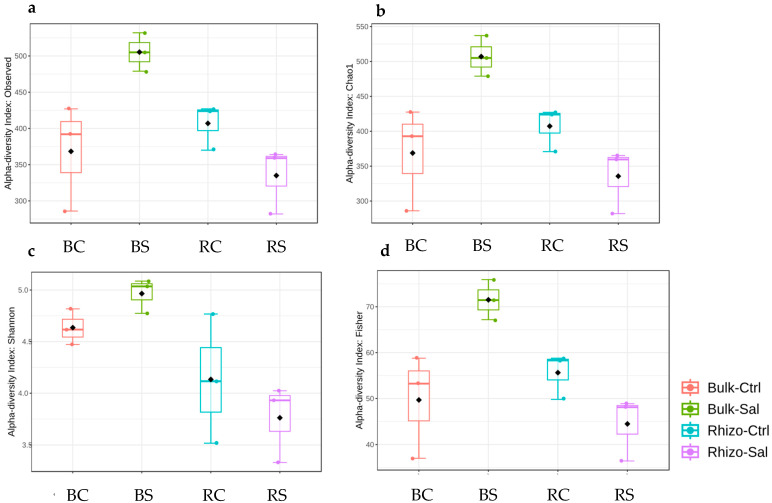
Box-and-whisker plots illustrate the alpha diversity indices between treatments for different soil types and circumstances. (**a**) The indices used are the *Observed* index (*p*-value: 0.012256; ANOVA F-value: 7.0649). (**b**) *Chao1* index (*p*-value: 0.012571; ANOVA F-value: 7.0011). (**c**) *Shannon* index (*p*-value: 0.021317; ANOVA F-value: 5.7599). (**d**) *Fisher* index (*p*-value: 0.010558; ANOVA F-value: 7.4478). The samples are classified as non-plant-associated bulk soil (BC, BS) and plant-associated rhizosphere soil (RC, RS) under both normal and saline conditions.

In the case of the non-associated plant mass, the microbiomes under normal conditions showed lower indices than their saline counterparts, indicating lower diversity Figure 3. Under normal conditions, the observed species richness was lower in (BC) compared to (RC). Under saline conditions, the observed species richness was higher in (BS) than in (RS) Figure 3a. The *Chao1* index, which measures species diversity, followed a similar pattern (*p*-value = 0.01, ANOVA *F*-value = 7.0649 Figure 3b). For the actual diversity indices, the *Shannon* index, which considers both richness and evenness, was higher in BC and BS than in RC and RS (*p*-value = 0.02, ANOVA F-value = 5.7599 Figure 3c). The *Fisher* index, another measure of diversity, was lower in BC than in RC under normal conditions but higher in BS and lower in R under saline conditions (*p*-value = 0.01, ANOVA F-value = 7.447 Figure 3d).

#### 3.3.2. Beta Diversity Assessment

**Figure 4:**
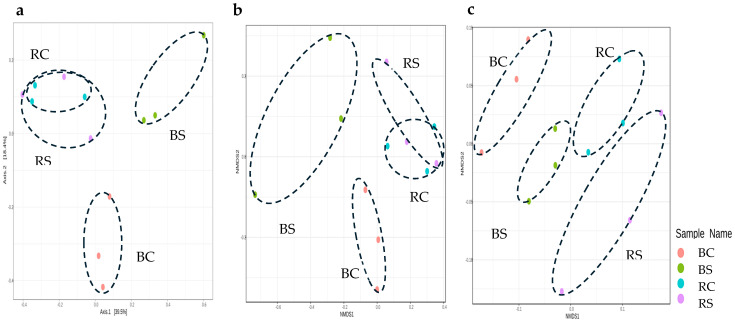
Ordination of beta diversity at the feature level using different methods: (**a**) PCoA ordination based on Bray–Curtis distances at the feature level. Statistical analysis was performed using PERMANOVA and resulted in an *F*-value of 3.3278, an *R*-squared value of 0.55145, and a *p*-value of 0.001. (**b**) Non-metric multidimensional scaling (NMDS) ordination based on Bray–Curtis distances at the trait level. Statistical analysis was performed using PERMANOVA and resulted in an *F*-value of 3.2784, an *R*-squared value of 0.55145, and a *p*-value of 0.001. The NMDS stress value is 0.080323. (**c**) PCoA ordering is based on Jensen–Shannon divergence at the trait level. Statistical analysis was performed using ANOSIM and resulted in an R-squared value of 0.39506 and a *p*-value of <0.009.

For beta diversity mapping Figure 4, Bray–Curtis distances showed significant clustering of the microbiome profiles of non-plant-associated soil (BC and BS) from the rhizosphere of wheat (RC and RS) presented in both Principle Coordinate Analysis (PCoA) ordination and non-metric multidimensional scaling (NMDS) ordination (PERMANOVA; *F*-value of 3.3278, *R*-squared = 0.55145, *p*-value = 0.001); and (PERMANOVA; NMDS stress value = 0.080323, *R*-squared = 0.55145, *p*-value of 0.001) Figure 4a,b. However, there was an overlapping grouping for the rhizosphere under both saline and normal conditions. Similarly, there was significant grouping into four clear clusters for the microbiome profile based on soil types and stress conditions. The latter was represented by the PCoA order based on Jensen–Shannon divergence (*ANOSIM*, *R*-squared = 0.39506 and *p*-value of <0.009 Figure 4c).

#### 3.3.3. Core Microbiome Analysis

**Figure 5:**
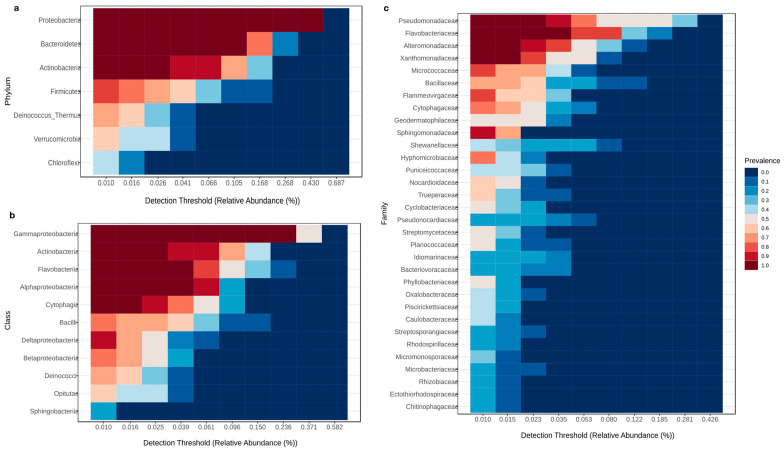
Heatmap showing the core microbiome of the wheat rhizosphere studied under both saline and normal conditions. Red indicates increased abundance, while blue indicates decreased abundance. Thresholds of 10% for prevalence and 0.1% for relative abundance were used at phylum level (**a**), class level (**b**), and family level (**c**).

Inspection and investigation of the major microbial taxa in the wheat microbiome under a variety of stressors and soil types emphasized the importance of these key microbial families in maintaining the stability and functionality of the microbial community. Analysis of the core microbiome was performed with a sample prevalence threshold of 20% and a relative abundance threshold of 0.01%. The results showed that the predominant taxa in all samples were *Proteobacteria* (*Xanthomonadaceae*, *Pseudomonadaceae*, and *Alteromonadaceae*), *Actinobacteria*, and *Bacteriodetes* (*Flavobacteriaceae*), each with a prevalence value of 1. In addition, several other families, including *Sphingomonadaceae*, *Micrococcaceae*, and *Flammeovirgaceae*, have high prevalence values, indicating their widespread occurrence in the dataset Figure 5a–c).

### 3.4. Correlation Analysis

This analysis helped to identify key species and predict ecological interactions within the microbial community, highlighting the diversity and dynamics within the microbiota. This enabled predictions to be made about patterns that are co-occurring or mutually exclusive within our microbial community. The results of Spearman rank correlation analysis at the phylum level between different bacterial taxa in soil and rhizosphere under normal and saline conditions are shown in Table S1. The correlations, ranging from −0.7251 to 0.9218, indicate the strength and direction of relationships between pairs of taxa.

Positive correlations indicate co-occurrence, i.e., when the abundance of one taxon increases, the abundance of the other taxon also increases, while negative correlations indicate an inverse relationship. In particular, strong positive correlations were found between *Aquificae* and *Synergistetes* (0.9218, *p* < 0.0001), *Spirochaetes* and *Synergistetes* (0.8656, *p* < 0.001), and *Tenericutes* and unclassified bacteria (0.903, *p* < 0.0001). Conversely, *Aquificae* and *Bacteroidetes* (−0.7251, *p* = 0.0076) and *Deinococcus_Thermus* and *Thermi* (−0.7251, *p* = 0.0076) showed strong negative correlations, indicating a possible competitive relationship or different ecological niches.

The results of the same analysis between bacterial families were also displayed at the family level. The correlations, which are all statistically significant (*p* < 0.000001), range from −0.9441 to 0.9901 Table S2. Several families showed strong positive correlations, such as *Aquificaceae* and *Synergistaceae* (0.9826), *Clostridiales family XVII Incertae Sedis* and *Holophagaceae* (0.9901), also *Nitriliruptoraceae* and *Thioalkalispiraceae* (0.977). These results indicate that the frequency of these family pairs tended to increase or decrease together under the conditions studied. Conversely, there were notable negative correlations between *Caldithrix* and *Nitriliruptoraceae* (−0.9413) and *Rhizobiaceae* and *Trueperaceae* (−0.9371), suggesting that the presence of one taxon may suppress the other.

### 3.5. Differential Abundance Profiling (DAP)

Multifactor analysis (*MaAsLin2* analysis; negative binomial model NeBIN) was applied at the family, genus, and species level to determine the signature of each microbial niche (to identify the unique microbiota of each environment). This information allowed us to identify the bacterial species with a significantly different abundance under different conditions.

a. Microbial recruiting:

Plant–environment interaction deals with the plant–microbial interaction due to the association of plants either under normal conditions or under salinity stimulation between the soil (BC/ RC) and the microbiota of the wheat rhizosphere (BS/RS).

1 Wheat microbial recruitment under normal conditions: BC vs. RC:

There were significant differences in the diversity and quantity of bacterial taxa between the topsoil (BC) and the wheat rhizosphere (RC) under normal conditions. Significant recruitment of certain phyla was observed in the wheat rhizosphere. There was a significant decrease in *Actinobacteria*: 20 taxa were present in the main soil (BC), but only one taxon in the rhizosphere soil (RC). *Proteobacteria* were also less abundant in the RC, with 11 species compared to 16 in the BC. *Bacteroidetes* showed a concomitant decline, with four taxa in BC compared to only two in RC. However, there was an influence on the marked presence of certain taxa. *Firmicutes*, *Nitrospirae,* and *Planctomycetes* were the main bacterial phyla contributing to the ecological functions of the soil ecosystem (BC), while *Tenericutes, Verrucomicrobia,* and *Caldithrix* bacteria were particularly well represented in the rhizosphere (RC). These results are from a multifactor analysis with a negative binomial model performed with the *MaAsLin2* tool (FDR cutoff of 0.05 and a Log2FC threshold > 2).

2 Wheat microbial recruitment under saline conditions: BS vs. RS:

Analysis of the bacterial taxa in the wheat rhizosphere (RS) compared to the topsoil (BS) under saline conditions revealed that *Actinobacteria* were significantly more abundant in the topsoil (18 taxa) than in the saline rhizosphere (only two taxa) Table 2. The *Bacteroidetes* were also slightly less present in the RS with one taxon, whereas they were represented by two taxa in the BS. In contrast, the recruitment of *Proteobacteria* followed an opposite trend, with a higher prevalence of 15 species in the rhizosphere soil under saline conditions, compared to only nine taxa in the main soil. *Spirochaetes* were represented by one taxon only in the topsoil, while *Fibrobacteres*, *Firmicutes,* and *Verrucomicrobia* were each represented by one or two taxa exclusively in the saline rhizosphere soil (RS). These results are from a multifactorial analysis with a negative binomial model performed with the *MaAsLin2* tool (FDR cutoff of 0.05 and a Log2FC threshold > 2).

b. Salinity-Induced Microbial Shifts:

The study showed changes in the microbial community of the wheat rhizosphere under saline irrigation compared to normal irrigation (RC versus RS). Thus, the microbial change in the wheat rhizosphere was investigated by salinity stimulation.

*Proteobacteria* showed the largest taxonomic number, especially under saline conditions, with 24 taxa compared to 8 taxa under normal conditions Table 3. Conversely, *Actinobacteria* showed a greater taxonomic number under normal conditions (7 taxa) than under saline conditions (1 taxon). *Bacteroidetes*, on the other hand, showed a higher taxonomic diversity (5 taxa) under salinity than under normal conditions. *Firmicutes* and *Nitrospinae* were only represented under saline conditions with three and one taxon, respectively, while Planctomycetes and *Spirochaetes* were only detected under normal conditions with one taxon each. This underlines the salinity tolerance of the taxa belonging to the *Proteobacteria*, *Firmicutes,* and *Nitrospinae*. These results are from a multifactor analysis with a negative binomial model performed with the *MaAsLin2* tool (FDR cutoff of 0.05 and a Log2FC threshold > 2).

c. Microbial signature:

*Idiomarinaceae, Rheinheimera, Halomonas,* and *Pseudomonas* (a strain of *Proteobacteria*), together with *Gracilibacillus* (a strain of *Firmicutes bacilli*), were consistently identified as microbial signatures in the rhizosphere microbiome under salt stress; these microbial signatures showed significantly greater changes than their counterparts under normal conditions. These results are from a multifactorial analysis with a negative binomial model performed with the *MaAsLin2* tool (FDR cutoff of 0.05 and a Log2FC threshold > 2).

## 4. Discussion

Metagenomics has transformed our understanding of plant–microbe interactions by highlighting the critical role of microbial communities in improving plant health and resilience. These communities contribute to nutrient uptake, stress tolerance, and disease suppression, while metagenomic analysis enables the identification of genes involved in nutrient cycling and growth stimulation. In saline environments, the rhizosphere microbiome is critical for maintaining plant productivity as the first line of plant defense.

### 4.1. Assessment of Abundance Count Data

In this study, the relative abundance of *Proteobacteria* (*Pseudomonadales_Pseudomonadaceae* and *Alteromonadales_Alteromonadaceae*), *Bacteroidetes,* and *Firmicutes* were shown to be high in the rhizosphere of wheat under salinity. Recent studies have emphasized the adaptation of microbial communities, especially the phyla of *Bacteroidetes*, *Firmicutes*, *Proteobacteria,* and *Actinobacteria*, to saline conditions [32]. In addition, improved salt tolerance in wheat, rice, and barley has been correlated with the phyla *Bacteroidetes* and *Firmicutes* improving their physiological properties [33,34].

### 4.2. Alpha and Beta Diversity Analysis

Salinity significantly increased diversity in the soil but decreased in the rhizosphere. Our study revealed that microbial taxonomic alpha diversity was significantly lower in the rhizosphere of wheat than in soil, suggesting different microbial communities in these two environments. Along with the analysis of alpha diversity, the rhizosphere forced a restriction of microbial richness, possibly due to selection pressure from plant roots, while salinity promoted a more complex microbial community structure in the topsoil [35,36]. The rhizosphere may harbor a specialized microbial assemblage that contributes more to functional than taxonomic diversity [37]. The effects of drought stress were found to increase both alpha and beta diversity in the wheat microbiome [38].

The analysis of beta diversity showed a significant clustering of the rhizospheric soil from that of the main soil. This separation was evident in both PCoA ordering and non-metric multidimensional scaling (NMDS), indicating different microbial community structures between these soil types and stress conditions. The beta diversity result emphasizes that selection enrichment for functional traits, rather than taxonomic composition, maybe the primary reason for the observed differences in beta diversity between bulk and rhizosphere soils under normal and saline conditions. The pronounced separation of microbiome profiles observed in our study is consistent with previous research demonstrating the influence of environmental factors on soil and rhizosphere microbial communities [38].

### 4.3. Consistent Core Microbiome Communities

The consistent presence of some microbial taxa in the rhizosphere in different plant types has been explained by the fact that certain core taxa play a fundamental role in improving plant resistance and growth, even when the nature of the microbial communities’ changes. *Proteobacteria*, *Bacteroidetes,* and *Actinobacteria* were found to be strategic groups of plant growth-promoting rhizobacteria (PGPR) that significantly promote plant growth through their involvement in nutrient cycling and phytohormone production.

### 4.4. Stability of Microbial Communities

The study of microbial communities in bulk and rhizosphere soils in both normal and salinized environments revealed considerable coexistence and competitive interactions. Studies suggest that both the diversity of interactions and the ecological role of individual taxa are critical for maintaining the stability of microbial communities, especially in dynamic environments where disturbances are common [39,40]. Microbial communities with balanced cooperative, competitive, and antagonistic interactions have been found to have higher values.

### 4.5. Variation in Bacterial Abundance Across Treatments

*Proteobacteria* were the largest strain present in the main and rhizosphere in all treatments in our study. The abundance varied depending on the treatment. Although *Proteobacteria* were less abundant in the normally irrigated soil (RC), they were recruited more in the rhizosphere of plants when they were under salt stress (RS). This strain was also represented as a microbial signature in (RS) by *Idiomarinaceae*, *Rheinheimera*, *Halomonas,* and *Pseudomonas* (*Proteobacteria* strain), which are known for their salt tolerance. For example, *Halomonas* species have been shown to mitigate the negative effects of salinity in purple basil (*Ocimum basilicum* L.) by increasing proline content and promoting osmotic regulation [41]. Similarly, they have been found to improve the growth of chia (*Salvia hispanica* L.) seedlings under saline conditions [42].

In addition, *Halomonas* species have shown the potential to improve the salt tolerance of crops by modulating osmotic balance and increasing nutrient availability in saline soils [43]. A study has shown that inoculation with *Pseudomonas* and other PGPR significantly improves the growth of wheat under salt stress by increasing indole-3-acetic acid (IAA) content and promoting root elongation [44]. In addition, *Pseudomonas* strains have been reported to produce *1-aminocyclopropane-1-carboxylate* (ACC) deaminase, which helps to curb ethylene production under stress, thereby promoting better growth and stress tolerance in tomato [45,46]. In addition, they improve the antioxidant capacity of plants and help them deal with oxidative stress triggered by high salinity [47].

The strain of *Proteobacteria* not only contributes to supporting plant growth through the availability of nutrients but also triggers the upregulation in genes for phytohormones (IAA, auxins, and cytokinins) in wheat under salt stress [48,49,50]. *Actinobacteria* and *Bacteroidetes* phyla were similarly predominant and basic in all treatments but somehow less abundant in the rhizosphere under both normal and saline irrigation (RC and RS) compared to their counterparts. Studies on legumes such as *Pisum sativum* L. and *Cicer arietinum* L. have shown that *Actinobacteria* improve nutrient availability and promote growth by modulating hormone levels [51].

*Firmicutes* were remarkably abundant in the rhizosphere of wheat exposed to saline irrigation. *Firmicutes-Gracilibacillus* were represented as a microbial signature in (RS), which was identified as a potential PGPR and has properties that promote plant growth under saline conditions [52]. These bacteria can also boost the production of osmoprotectants that help stabilize cell functions under salt stress [53,54]. *Fibrobacteres* have also been observed to exhibit higher fold change in RS than their counterpart in BS. Halotolerant *Fibrobacteres*, known for their cellulolytic abilities, improve the availability of nutrients in saline soils [55]. In addition, their presence improved the agronomic properties of rice [56].

The *Tenericutes* strain and *Caldithrix* (unclassified bacterium) were found in the rhizosphere of wheat only under normal irrigation. This indicates that the wheat genotype used in our study can instinctively recruit some PGPR bacterial strains that promote plant growth under normal conditions. *Tenericutes* have been shown to be able to produce *indole-3-acetic acid* (IAA), an important plant hormone that promotes branching and root elongation [57]. *Caldithrix* is able to synthesize organic acids—such as citric acid and malic acid, which can improve nutrient availability in the rhizosphere [58]—in addition to producing volatile organic compounds (VOCs) that play a role in plant signaling and stress response, potentially increasing the plant’s resistance to abiotic stresses such as drought and salinity [59,60].

*Mycoplasmas*, a genus within the *Tenericutes* phylum, can influence the immunity and health of plants despite their pathogenic potential. These bacteria are known to interact with plant systems and potentially trigger immune responses. For example, the presence of mycoplasmas can trigger systemic acquired resistance (SAR) in plants, which improves their ability to resist subsequent attacks by pathogens [61]. This immune modulation could be related to the production of signaling molecules that activate defense mechanisms in plants, thus improving their resistance to various stress factors. In addition, mycoplasmas can alter the composition of the rhizosphere microbiome, which plays a crucial role in plant health. By promoting beneficial microbial communities, *Mycoplasma* can improve nutrient availability and uptake, thus supporting plant growth [62].

The strain *Verrucomicrobia* (*Opitutae*) was found to be present in the rhizosphere of wheat under both irrigation methods (RC and RS). *Verrucomicrobia* is known to produce a variety of extracellular enzymes such as cellulases, hemicellulases, and ligninases that facilitate the degradation of plant biomass and organic matter, thereby releasing essential nutrients such as nitrogen, phosphorus, and potassium for plant uptake [63,64].

## 5. Conclusions

Metagenomic studies of the rhizosphere of wheat not only highlight the diversity and potential benefits of microbes but also emphasize the need for innovative approaches to benefit from unculturable bacteria to resist salt stress. The phyla *Proteobacteria*, *Actinobacteria*, *Bacteroidetes*, *Firmicutes*, *Verrucomicrobia*, and *Fibrobacteres* represent the microbial profile of the wheat rhizosphere under different irrigation conditions. Among these bacteria, the bacterial phyla *Proteobacteria*, *Firmicutes*, and *Verrucomicrobia* increased in the salt-treated soils, while *Actinobacteria* and *Bacteroidetes* decreased after salt treatment. *Idiomarinaceae*, *Rheinheimera*, *Halomonas*, *Pseudomonas* (phylum *Proteobacteria*), and *Gracilibacillus* (phylum *Firmicutes Bacilli*) were significantly more represented in the rhizosphere microbiome under saline conditions than their counterparts under normal conditions. The collective action of these bacterial phyla in the rhizosphere not only improves nutrient availability but also induces systemic resistance in the plants, enhancing their ability to cope with abiotic stress factors such as salinity in this study.

This study highlights the importance of innovative strategies to take advantage of non-culturable microbes, possibly through synthetic biology and gene expression in culturable hosts, to ultimately translate metagenomic discoveries into pragmatic improvements in wheat growth. Identification of key microbial taxa and their metabolic contribution will enable the development of tailored microbial inoculants to increase wheat resilience under salt stress. The use of beneficial microorganisms in agricultural systems will reduce dependence on pesticides and improve soil health for sustainable agriculture.

## Figures and Tables

**Table 1:** Operational taxonomic unit (OTU) annotation statistics, using the Greengenes database, illustrating the number of OTUs identified, the counts per sample, and the comparison between matched metadata and the OTU table for all experimental conditions.

Type	Counts
OTU annotation:	GreengenesID
OTU number:	1110
OTUs with ≥2 counts:	752
Number of experimental factors with replicates:	3 [discrete: 3 continuous: 0]
Total read counts:	4,526,902
Average counts per sample:	377,241
Maximum counts per sample:	796,235
Minimum counts per sample:	141,355
Number of sample names matched (metadata vs. OTU table):	12

**Table 2:** Wheat microbial recruitment describes the plant–microbial interactions that significantly increased in abundance under salinity. Revealing a distinct microbial signature in the RS (Rhizosphere under Salinity) condition compared to BS (Bulk soil under saline conditions). Multifactor analysis using the *MaAsLin2* tool with a negative binomial model, with significance determined by an FDR cutoff of 0.05 and a Log2FC threshold of 2.

	Plant–Microbial Interaction (Microbial Recruitment)					
No.	Phylum (Class)	Taxa	Log2FC	Estimated BS	Fold Change	Percentage Increase (%)
1	*Proteobacteria* (*DeltaProteobacteria*)	*Sandaracinaceae*	3.37	0.097	10.34	933.88
2	*Proteobacteria* (*GammaProteobacteria*)	*Halomonas_xianhensis*	3.83	0.07	14.22	1322.15
3		*Legionella*	3.64	0.08	12.47	1146.66
4		*Thermomonas*	3.58	0.084	11.96	1095.88
5		*Azotobacter_armeniacus*	3.28	0.103	9.71	871.36
6		*Rhizobacter*	3.14	0.113	8.82	781.52
7		*Pseudomonas_sagittaria*	2.87	0.137	7.31	631.06
8		*Rhizobacter_fulvus*	2.78	0.145	6.87	586.85
9		*Halomonas_daqiaonensis*	2.43	0.185	5.39	438.89
10		*Rheinheimera*	2.35	0.196	5.1	409.82
11		*Pseudomonas_azotifigens*	2.34	0.198	5.06	405.88
12	*Proteobacteria* (*AlphaProteobacteria*)	*Devosia_terrae*	2.77	0.147	6.82	582.11
13		*Altererythrobacter_sp_*	2.22	0.215	4.66	366.24
14		*Novosphingobium*	2.11	0.231	4.32	331.69
15		*Altererythrobacter*	2.09	0.234	4.26	326.27
16	*Actinobacteria*	*Saccharothrix_hoggarensis*	3.46	0.091	11	1000.43
17		*Saccharothrix*	4.03	0.061	16.34	1533.62
18	*Firmicutes* (*Bacilli*)	*Gracilibacillus_ureilyticus*	3.26	0.104	9.58	858.35
19	*Fibrobacteres* (*Fibrobacteria*)	*Fibrobacteraceae*	2.29	0.205	4.89	388.93
20	*Bacteroidetes* (*Flavobacteriia*)	*Ulvibacter*	2.81	0.143	7.01	601.28
21	*Verrucomicrobia* (*Opitutae*)	*Opitutaceae*	2.11	0.231	4.32	331.69
22		*Puniceicoccaceae*	2.24	0.212	4.72	372.43

**Table 3:** Salinity impact on microbial traits, with a microbial signature showing significant increases in abundance in the rhizosphere under salinity (RS) compared to normal conditions (RC). Multifactor analysis using the *MaAsLin2* tool with a negative binomial model, with significance determined by an FDR cutoff of 0.05 and a Log2FC threshold of 2, determined using an FDR cutoff of 0.05 and a Log2FC threshold of 2.

Salinity-Induced Microbial Shifts						
No.	Phylum (Class)	Taxa	Log2FC	RC	Fold Change (FC)	Percentage Increase of RS over RC (%)
1	*Proteobacteria (GammaProteobacteria)*	*Idiomarinaceae*	6.11	0.014477938	69.07060714	6807.060714
2		*Microbulbifer*	3.61	0.081899588	12.21007367	1121.007367
3		*Rheinheimera*	3.43	0.092782723	10.77786861	977.7868615
4		*Halomonadaceae*	3.13	0.114228931	8.75434961	775.434961
5		*Marinobacter_sp_*	3.11	0.115823508	8.633825892	763.3825892
6		*Halomonas*	2.98	0.126744935	7.889861636	688.9861636
7		*Marinobacter*	2.8	0.143587294	6.964404506	596.4404506
8		*Pseudomonas_sp_*	2.29	0.204475515	4.890561111	389.0561111
9		*Pseudomonas_sagittaria*	2.17	0.22221067	4.500233939	350.0233939
10	*Firmicutes (Bacilli)*	*Gracilibacillus*	4.45	0.045752678	21.85664411	2085.664411
11		*Gracilibacillus_kekensis*	3.73	0.075362989	13.26911273	1226.911273
12		*Bacillaceae*	2.04	0.243163737	4.112455307	311.2455307
13	*Bacteroidetes (Cytophagia)*	*Nafulsella*	3.72	0.07588718	13.17745628	1217.745628
14	*Bacteroidetes (Flavobacteriia)*	*Salegentibacter*	3.71	0.076415017	13.08643294	1208.643294
15		*Salinimicrobium_marinum*	3.45	0.091505356	10.92832205	992.8322054
16		*Psychroflexus*	2.86	0.137738139	7.260153243	626.0153243

## Data Availability

The datasets generated during the current study are available in the NCBI repository, under the Bioproject number PRJNA1190448, available through the web link https://www.ncbi.nlm.nih.gov/sra/PRJNA1190448 (accessed on 1 June 2024). The corresponding accession numbers for this submission are: SRR31504559, SRR31504564, SRR31504562, SRR31504561, SRR31504558, SRR31504555, SRR31504563, SRR31504560, SRR31504557, SRR31504554, SRR31504556, SRR31504553.

## Supplementary Materials

The following supporting information can be downloaded at https://www.mdpi.com/article/10.3390/plants14071033/s1. Table S1: Correlation analysis at the phylum level under different soil and salinity conditions. Spearman’s rank correlation coefficients and associated *p*-values highlight co-occurrence and inverse relationships between phyla across bulk and rhizosphere soils. Table S2: Family-level correlation analysis under different soil and salinity conditions. Spearman’s rank correlation coefficients and *p*-values provide insights into interactions between bacterial families across experimental conditions.
