# Supplementary material for: Characterizing Wheat Rhizosphere Bacterial Microbiome Dynamics Under Salinity Stress: Insights from 16S rRNA Metagenomics for Enhancing Stress Tolerance
Source: Plants (Basel). 2025 Mar 26;14(7):1033. doi: 10.3390/plants14071033 (PMC11990312; doi:10.3390/plants14071033)
Supplement: Supplementary file 1 [file plants-14-01033-s001.zip › plants-3490451-supplementary.pdf]

Supplementary Table S1: Correlation analysis at the phylum level under different soil and salinity conditions. Spearman's rank correlation coefficients and associated p-values highlight co-occurrence and inverse relationships between phyla across bulk and rhizosphere soils.

**Taxon1**	**Taxon2**	**Correlation**	**P-value**
Actinobacteria	*Nitrospirae*	0.8049	0.0016
Aquificae	*Bacteroidetes*	-0.7251	0.0076
Aquificae	*Dictyoglomi*	0.8086	0.0015
Aquificae	*Ignavibacteriae*	0.7852	0.0025
Aquificae	*Spirochaetes*	0.8339	7e-04
Aquificae	*Synergistetes*	0.9218	0
Bacteroidetes	*Proteobacteria*	0.8322	8e-04
Bacteroidetes	*Thermi*	0.716	0.0088
Bacteroidetes	*Verrucomicrobia*	0.7832	0.0026
Cyanobacteria	*Firmicutes*	0.7391	0.006
Deinococcus_Thermus	*Dictyoglomi*	0.8032	0.0017
Deinococcus_Thermus	*Firmicutes*	0.7762	0.003
Deinococcus_Thermus	*Ignavibacteriae*	0.7114	0.0095
Deinococcus_Thermus	*Thermi*	-0.7251	0.0076
Dictyoglomi	*Ignavibacteriae*	0.7701	0.0034
Dictyoglomi	*Synergistetes*	0.7217	0.008
Gemmatimonadetes	*Synergistetes*	0.733	0.0067
Ignavibacteriae	*Nitrospirae*	0.7679	0.0035
Ignavibacteriae	*Spirochaetes*	0.753	0.0047
Ignavibacteriae	*Synergistetes*	0.8536	4e-04
Proteobacteria	*unclassified_bacteria*	0.8161	0.0012
Proteobacteria	*Verrucomicrobia*	0.8112	0.0014
Spirochaetes	*Synergistetes*	0.8656	3e-04
Tenericutes	*unclassified_bacteria*	0.903	1e-04
Tenericutes	*Verrucomicrobia*	0.7747	0.0031
Thermi	*Verrucomicrobia*	0.716	0.0088
unclassified_bacteria	*Verrucomicrobia*	0.8792	2e-04

Supplementary Table S2: Family-level correlation analysis under different soil and salinity conditions. Spearman's rank correlation coefficients and p-values provide insights into interactions between bacterial families across experimental conditions.

**Taxon1**	**Taxon2**	**Correlation**
Acidimicrobiaceae	Nitriliruptoraceae	0.9397
Alcanivoracaceae	Deinococcaceae	0.965
Aquificaceae	Synergistaceae	0.9826
Aurantimonadaceae	Desulfovibrionaceae	0.9441
Aurantimonadaceae	Paenibacillaceae	0.9441
Caldithrix	Nitriliruptoraceae	-0.9413

Caulobacteraceae	Ectothiorhodospiraceae	-0.9441
Clostridiales_Family_XVII__Incertae_Sedis	Holophagaceae	0.9901
Cyclobacteriaceae	Methylobacteriaceae	-0.9441
Dictyoglomaceae	Intrasporangiaceae	0.9652
Erythrobacteraceae	Sphaerobacteraceae	-0.9441
Euzebyaceae	Flammeovirgaceae	-0.9499
Euzebyaceae	Paenibacillaceae	0.9572
Geodermatophilaceae	Iamiaceae	0.9752
Geodermatophilaceae	Jiangellaceae	0.9507
Geodermatophilaceae	Nitriliruptoraceae	0.9397
Geodermatophilaceae	Thioalkalispiraceae	0.9422
Halomonadaceae	Pelobacteraceae	0.9574
Hyphomicrobiaceae	Puniceicoccaceae	0.9371
Iamiaceae	Jiangellaceae	0.9535
Iamiaceae	Nitriliruptoraceae	0.971
Iamiaceae	Planctomycetaceae	0.9681
Iamiaceae	Thioalkalispiraceae	0.9484
Iamiaceae	Thiotrichaceae	0.9325
Jiangellaceae	Planctomycetaceae	0.9648
Kofleriaceae	Micromonosporaceae	0.9352
Nitriliruptoraceae	Planctomycetaceae	0.9539
Nitriliruptoraceae	Thioalkalispiraceae	0.977
Planococcaceae	Polyangiaceae	-0.9441
Rhizobiaceae	Trueperaceae	-0.9371
Thioalkalispiraceae	Thiotrichaceae	0.9457
